# Long-term responders to trastuzumab monotherapy in first-line HER-2+ advanced breast cancer: characteristics and survival data

**DOI:** 10.1186/s12885-019-6105-3

**Published:** 2019-09-10

**Authors:** Sabine Schmid, Dirk Klingbiel, Stefan Aebi, Aron Goldhirsch, Christoph Mamot, Elisabetta Munzone, Franco Nolè, Christian Oehlschlegel, Olivia Pagani, Bernhard Pestalozzi, Christoph Rochlitz, Beat Thürlimann, Roger von Moos, Patrik Weder, Khalil Zaman, Thomas Ruhstaller

**Affiliations:** 1 Breast Center St. Gallen, Kantonsspital, Rorschacherstrasse 95, 9007 St. Gallen, Switzerland; 2 Swiss Group for Clinical Cancer Research (SAKK), Berne, Switzerland; 3 Cancer Center, Lucerne Cantonal Hospital, Lucerne and University of Bern, Bern, Switzerland; 4 Department of Oncology, European Institute of Oncology (IEO), Milan, Italy; 5 Department of Oncology and Haematology, Kantonsspital Aarau, Aarau, Switzerland; 6 Division of Medical Senology, European Institute of Oncology (IEO), Milan, Italy; 7 Formerly Institute of Pathology, Kantonsspital St. Gallen, St. Gallen, Switzerland; 8 Breast Unit and Institute of Oncology of Southern Switzerland, Ospedale Regionale Bellinzona e Valli and Geneva University Hospitals, Bellinzona, Switzerland; 9 Department of Oncology, University Hospital Zürich, Zürich, Switzerland; 10 Department of Oncology, University Hospital Basel, Basel, Switzerland; 11 Department of Oncology, Kantonsspital Graubünden, Chur, Switzerland; 12 Breast Center CHUV, Department of Oncology, University Hospital CHUV, Lausanne, Switzerland; 13 Medizinische Fakultät, Universität Basel, Basel, Switzerland

**Keywords:** HER-positive breast cancer, Trastuzumab monotherapy, Long-term responders

## Abstract

**Background:**

The impact of HER2-targeted therapy alone followed by the addition of chemotherapy at disease progression (PD) versus upfront combination was investigated by the SAKK 22/99 trial. The aim of this exploratory analysis of the SAKK 22/99 trial was to characterize the specific subset of patients deriving long-term benefit from trastuzumab monotherapy alone and to identify potential predictive factors of long-term response.

**Methods:**

This is an unplanned post-hoc analysis of patients randomized to Arm A (trastuzumab monotherapy). Patients were divided in two groups: patients with durable clinical benefit from trastuzumab monotherapy and short-term responders without durable clinical benefit from trastuzumab monotherapy Univariate and multivariate analyses of clinical characteristics correlating with response duration was performed.

**Results:**

Eighty six patients were randomized in arm A, 24 patients (28%) were long-term responders and 62 (72%) were short-term responders with a 5y-overall survival (OS) of 54% (95% CI 31–72) and of 18% (95%CI 10–30), respectively. Absence of ER expression, absence of PgR expression and presence of visceral disease emerged as possible negative predictive factors for durable clinical benefit.

**Conclusion:**

Durable clinical benefit can be achieved with trastuzumab monotherapy in a subgroup of HER2-positive patients with advanced disease and it is predictive for longer OS. Further investigations of predictive biomarkers are necessary to better characterize this subgroup of patients and develop further de-escalating strategies.

**Trial registration:**

NCT00004935; first posted 27.01.2003, retrospectively registered.

**Electronic supplementary material:**

The online version of this article (10.1186/s12885-019-6105-3) contains supplementary material, which is available to authorized users.

## Background

About 15–20% of breast cancers overexpress HER2: in advanced breast cancer (ABC), HER2-targeted therapies significantly improve disease outcomes [1, 2]. Before the introduction of dual HER2-blockade (trastuzumab plus pertuzumab) in combination with chemotherapy [3] and trastuzumab emtansine (T-DM1) [4] as recommended first and second line therapy, respectively, trastuzumab in combination with chemotherapy was the standard of care. The impact of trastuzumab monotherapy followed by the addition of chemotherapy at disease progression (PD) versus upfront combination therapy was explored in the Swiss Group for Clinical Cancer Research (SAKK) 22/99 trial, a randomized phase III trial of sequential versus combination therapy in patients with HER2-positive ABC. The primary endpoint was time to progression after combination therapy (combination TTP). The recently published results [5], showed that both combination TTP and OS did not significantly differ between arms, suggesting that chemotherapy and its toxicity can be deferred, especially in patients with indolent, non-visceral disease. In particular, although the median TTP in the trastuzumab monotherapy arm was only 3.7 months, 6% of patients treated with single agent trastuzumab were still on treatment without PD after 2 years. This suggests that trastuzumab monotherapy may achieve long-term disease control in a subset of patients, in whom we can avoid upfront combination immuno-chemotherapy.

The aim of this unplanned analysis of the SAKK 22/99 trial was to characterize this specific subset of patients and to identify potential predictive factors of long-term response.

## Methods

SAKK 22/99 accrued patients from November 1999 to January 2013 [5]. This was a multicentre, prospective, non-blinded, randomized phase III trial. Patients with HER2-positive ABC were randomly assigned (1:1) to trastuzumab alone followed, at PD, by the combination with chemotherapy (Arm A) versus the upfront combination of trastuzumab and chemotherapy (Arm B). Chemotherapy could be stopped after six cycles in responding patients, trastuzumab was continued until progression.

Patients were stratified for HER2-ratio, adjuvant anthracyclines, estrogen/progesteron receptor (ER/PgR) status, line of treatment and institution. The primary trial endpoint was TTP on combined trastuzumab-chemotherapy (combination TTP) in both arms. The median follow-up was 77 months. SAKK 22/99 study design and methods have been previously described [5].

**Fig. 1:**
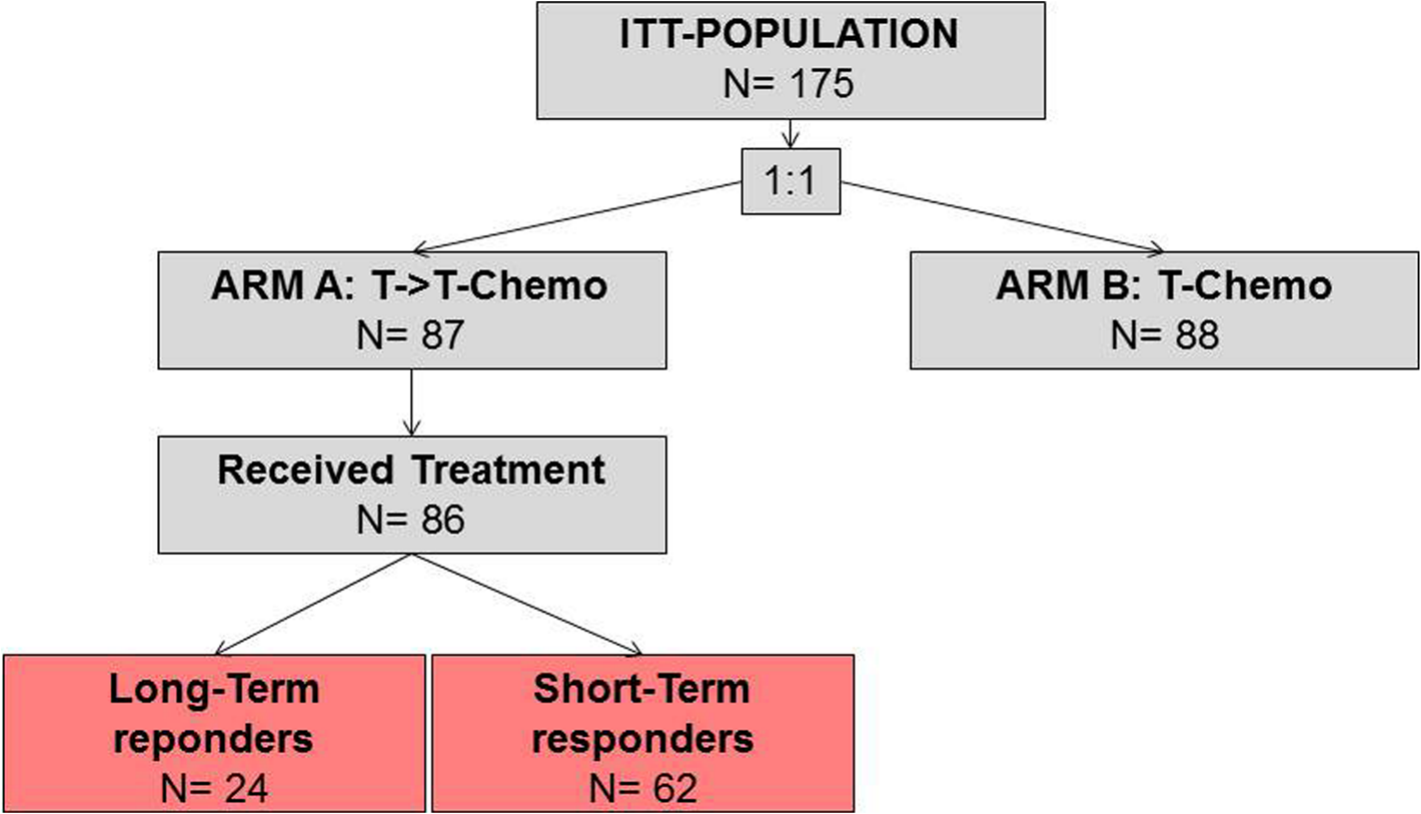
Flow-Chart

In this unplanned post-hoc analysis we focused on patients randomized to Arm A (trastuzumab monotherapy). We divided Arm A patients in two groups: long-term responders: patients with durable clinical benefit from trastuzumab monotherapy and short-term responders without clinical benefit to trastuzumab monotherapy (Fig. 1: Flow Chart). Durable clinical benefit was defined as complete response (CR), partial response (PR) or stable disease (SD) as best overall response that persisted for a minimum of 24 weeks. The cut-off of 24 weeks was chosen a priori based on the definition of durable clinical benefit commonly used in clinical trials [6, 7]. Twenty four weeks corresponds also well to the median response duration achieved with modern HER2-targeted therapies [8].

The primary outcome of interest for this study was OS, defined as time from randomization to death of any cause or censored at the last date the patient was known to be alive. To account for the immortal-time bias introduced by the definition of the groups with long or short clinical benefit we applied the landmark analysis method [7] for both the Kaplan-Meier method and uni- and multivariable Cox proportional hazards regression models. Standard descriptive measures such as median and interquartile range for continuous variables and absolute and relative frequencies for categorical variables were applied as appropriate and indicated in the text.

We analysed the following characteristics known to be associated with durable clinical benefit: visceral disease, ER/PgR expression, and the HER2:CEP17 ratio. Ki67 and tumour grade were not considered since not collected in the trial database.

*P*-values are two-sided and considered significant if < 0.05. No adjustment was made for multiple testing. Analyses were carried out using SAS v9.2 or the R software package (https://www.r-project.org) version 3.2 or later.

## Results

Of the 175 patients enrolled in the trial, 87 were randomised to receive trastuzumab monotherapy until PD and 86 ultimately received treatment according to protocol. Of these, 24 patients (28%) were long-term responders with durable clinical benefit and 62 (72%) were short-term responders according to the definition above (see Fig. 1).

Baseline characteristics such as age, prior endocrine therapy and ER/PgR expression were not different between the two groups when retrospectively analysed; this also holds true for laboratory values such as haemoglobin (Hb), white blood cell count (WBC) and alkaline phosphatase (ALP), which have been shown to be prognostic for OS in ABC [9]. The presence of visceral disease was the only variable correlated with durable clinical benefit, with only 46% of patients with visceral disease at time of randomisation being long-term responders compared to 76% in the short-term responders group (Fisher’s exact test *p* = 0.01) (Table 1: Baseline Characteristics of long-term versus short-term responders).

**Table 1:** Baseline Characteristics of long-term versus short-term responders

	HER2-long Responders (*N* = 24)	HER2-short Responders (*N* = 62)
Age (med, IQR)	57 years (48–67)	52 years (32–60)
Visceral disease (yes)	46%	76%
ER-Status (pos)	59%	60%
PgR-Status (pos)	48%	41%
FISH (med. Ratio, IQR)	4.8 (4.1–5.3)	4.7 (2.5–5.3)
ALP U/l (med, IQR)	74 (62–118)	103 (65–193)
Hb g/l (med, IQR)	130 (122–137)	134 (125–140)
WBC G/l (med, IQR)	5.8 (5.1–6.7)	6.8 (6.0–8.5)
Adjuvant endocrine treatment (yes)	33%	48%

When analysing the combination of possible negative predictive factors (NPF) such as absence of ER and PgR expression and presence of visceral disease and associating them with the duration of benefit from trastuzumab monotherapy, the proportion of long-term responders decreased with the presence of each additional NPF: 0 NPF 42%; 1 NPF 40%; 2 NPFs 35%; 3 NPFs 17%.

**Fig. 2:**
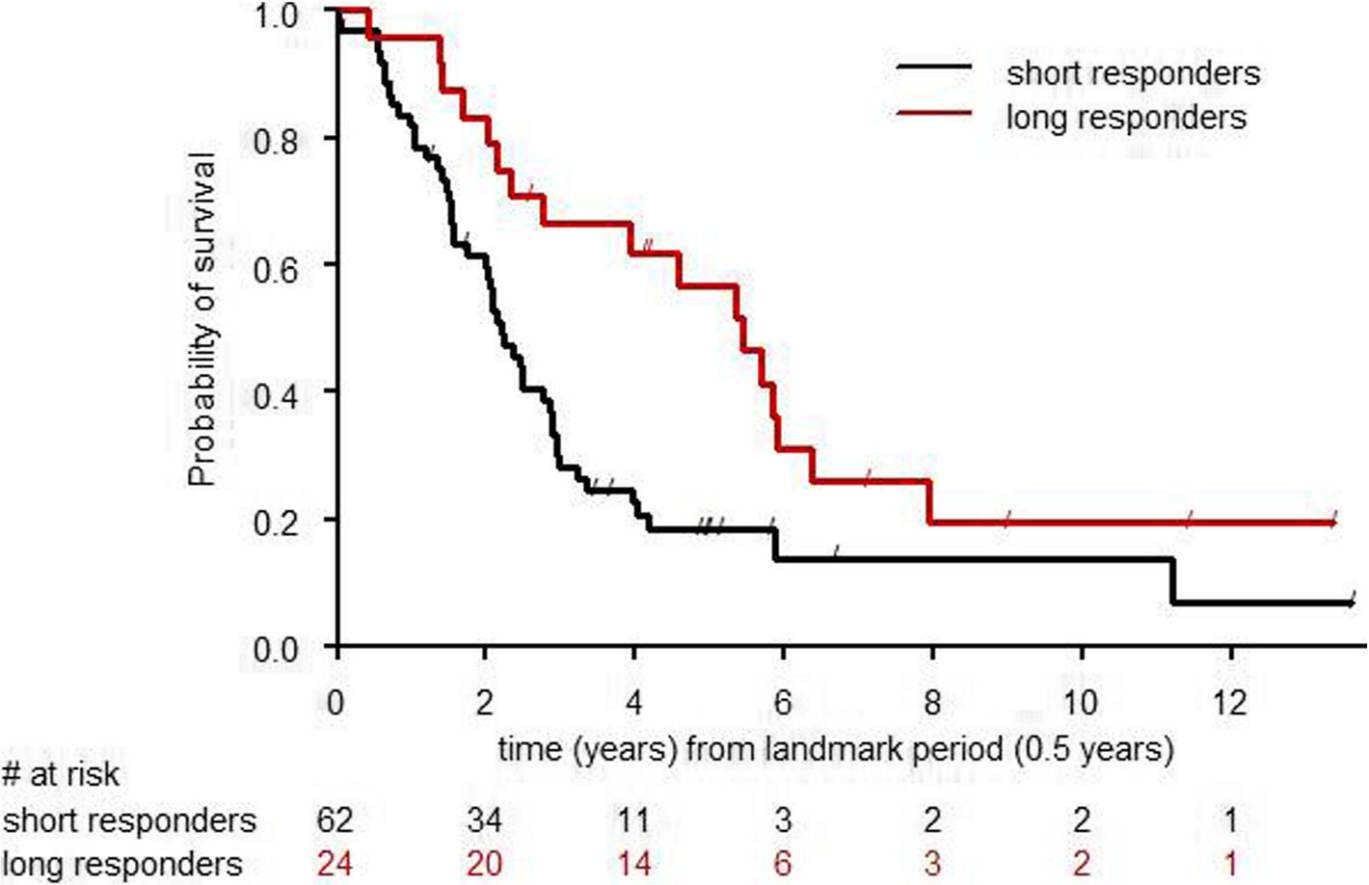
OS of long-term versus short-term responders

In a landmark analysis excluding patients who died within the first 6 months, the group of long-term responders to trastuzumab monotherapy showed a 5-year OS rate of 54% (95% CI 31–72) compared to only 18% (95% CI 10–30) among the short-term responders (log-rank *p* = 0.02) (Fig. 2: OS of long-term versus short-term responders).

In a multivariate analysis adjusted for visceral disease, durable clinical benefit from trastuzumab monotherapy remained significantly associated with OS (HR of 0.54, 95% CI 0.30–0.97, *p*-value: 0.04), suggesting long-term response to trastuzumab monotherapy correlates with long-term survival.

Regarding the degree of HER2 overexpression, we found a median FISH ratio of 4.8 (IQR 4.1–5.3) in long-term responders and of 4.7 (IQR 2.5–5.3) in short-term responders, respectively, showing a numerical, but not statistically significant difference in the first quartile. However, in the lowest quartile of all FISH ratios (≤3.4) there were only 3 (17%) long-term responders compared to 15 (83%) short responders.

**Fig. 3:**
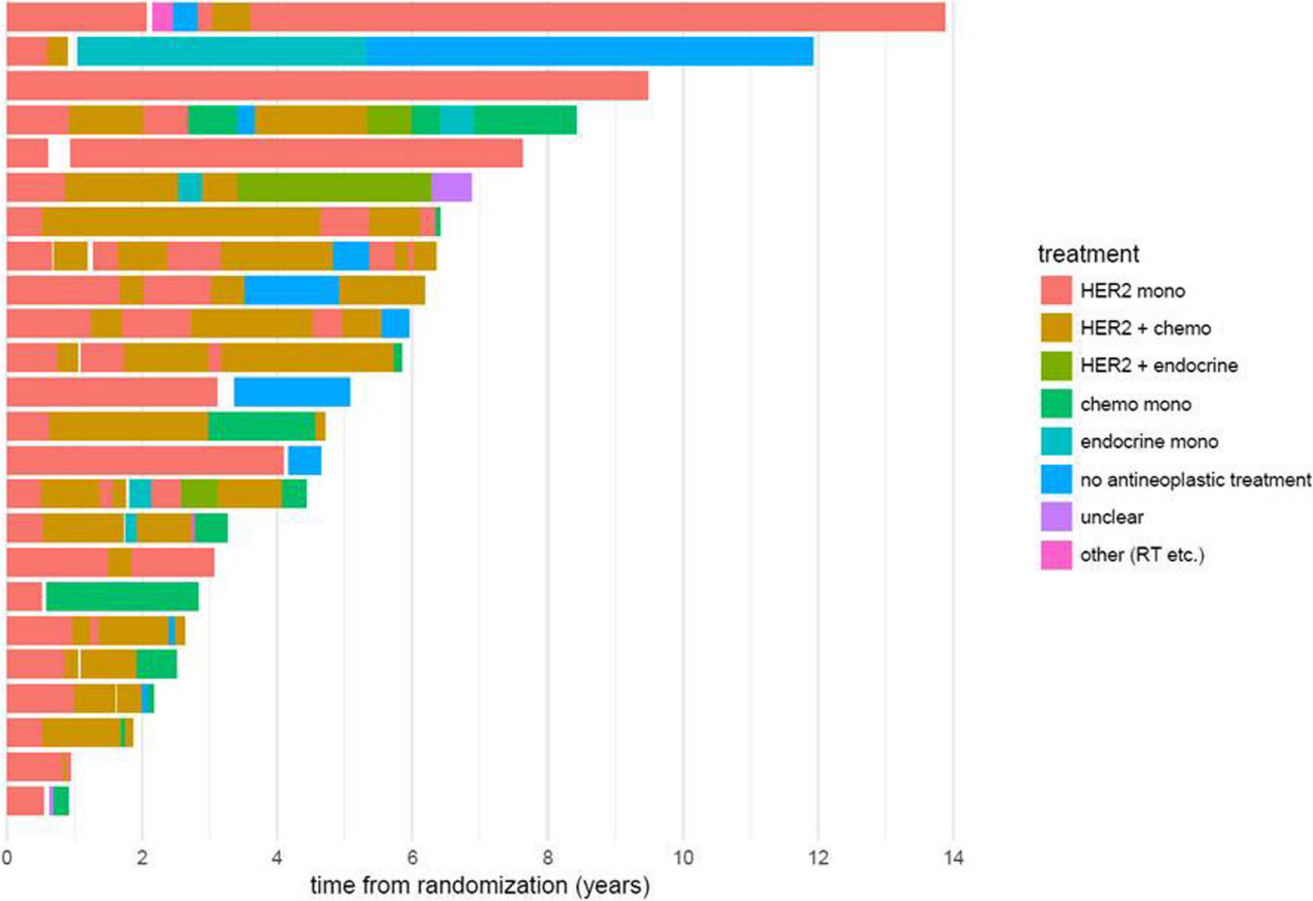
Patterns of care of long-term responders including follow-up treatment

Treatment patterns after PD in the group of long-term responders were very diverse. After trial treatment, patients received a median of 4 post-progression treatment lines, with some patients receiving up to 12 additional treatment lines. The majority of patients were treated with chemotherapy, either alone or in combination with HER2-directed treatments. Twenty-nine percent of patients also received one or several lines of endocrine treatment (monotherapy or in combination) in the course of their disease (Fig. 3: Patterns of care of long-term responders including follow-up treatment).

## Discussion

In this explorative analysis of the SAKK 22/99 trial we identified a subset of patients who derived long-term benefit from trastuzumab monotherapy and we show that long-term response is predictive for OS in a landmark analysis.

Current clinical research in HER2-positive ABC focuses on both upfront combination treatment approaches and de-escalating strategies. The triplet combination of trastuzumab, pertuzumab and chemotherapy as 1st-line treatment is considered the standard of care, showing a significant OS benefit of 15.7 months versus the double combination trastuzumab and chemotherapy in the CLEOPATRA trial [3]. But, as demonstrated in the SAKK 22/99 trial, a subset of patients may actually have long-term disease control with trastuzumab alone, sparing or at least postponing the toxicity of chemotherapy without compromising outcomes. Due to the lack of valid predictive biomarkers allowing early and effective identification of this specific subgroup of patients, in routine clinical practice patients are usually treated upfront with combination immuno-chemotherapy.

In this report, focusing specifically on patients treated with upfront trastuzumab monotherapy within a randomized clinical trial, we demonstrate that about a quarter of patients with advanced HER2-positive disease had at least 6 months disease control with single agent trastuzumab. This so-called durable clinical benefit to initial trastuzumab monotherapy was associated with an OS benefit compared to patients with short-term response. Potential factors associated with short-term response were the presence of visceral disease at randomization and numerically, but not statistically significant, potentially due to small numbers and availability in a subset of patients only, low HER2 FISH ratio. Visceral disease in combination with ER/PgR expression and possibly HER2 FISH ratio shows some association with the duration of treatment response. This is in line with previous reports of factors associated with long-term survivorship in patients with HER2-positive metastatic breast cancer [10, 11].

However, none of these parameters, alone or in combination, allows a highly reliable discrimination between the two patient groups and further investigation of predictive biomarkers is needed to improve the predictive value of such clinical factors.

Two other trials evaluated upfront HER2-targeted treatment alone followed by combination treatment versus upfront combination: the JO17360 trial assessed trastuzumab followed by trastuzumab and docetaxel at progression versus the upfront combination [12], whereas in the HERTAX trial trastuzumab followed by docetaxel alone at disease progression was compared to the upfront combination [13]. In both trials the upfront combination therapy was associated with superior OS compared to the sequencing approach achieving statistical significance only in the JO17360 trial. The conclusion from both trials was that their data do not support a sequential antibody-strategy. Interestingly, in the HERTAX trial a small subset of patients derived long-term benefit from trastuzumab alone: 16 and 4 patients (out of the 45 randomized in the sequential arm) were not progressing on trastuzumab monotherapy at 6 and 12 months, respectively. Unfortunately, the characteristics of these patients were not analysed in more detail. It would be interesting to pool all the three trials datasets to investigate and possibly validate predictive clinical factors for this subgroup of patients. Another more recent SAKK trial (22/10) investigated a similar treatment strategy by comparing the more active dual HER2-blockade alone versus upfront combination with chemotherapy both followed by T-DM1 at PD and first results were now presented at ESMO showing no impact on survival when deferring chemotherapy [14] and therefore support our results..

Our analysis clearly demonstrates that long-term response to trastuzumab monotherapy is predictive for OS with a 5-year OS of 54% in long-term responders versus only 18% in short-term responders. It would be of great clinical importance to prospectively characterize this good prognosis subgroup potentially de-escalate treatment and spare some patients the chemotherapy-related toxicities. This approach would also enhance cost-effectiveness as recently addressed by Nixon et al. [8].

Treatment patterns after PD in the group of long-term responders were very diverse: the majority of patients were treated with chemotherapy-based regimes. Thus, chemotherapy-related toxicity was significantly postponed due to the initial HER2-targeted monotherapy approach. However, the vast majority of patients received chemotherapy as well as endocrine treatments patients at some point in their disease course and therefore de-escalation of treatment in most cases has to be understood as a deferring approach**.** This approach has been already adopted in clinical practice mainly based on general principles usually used in the management of metastatic breast cancer and on clinical experience treating physicians [15].

This analysis has a number of limitations: First, this is an unplanned analysis with a relatively small sample size. Durable clinical benefit, though now often used in biomarker trials in the immunotherapy era, is not common hard endpoint in randomized trial. However, the 6 months cut-off is used for categorising clinical benefit when investigating efficacy endocrine treatment, another targeted therapy. Additional prognostic factors currently considered relevant, such as Ki67 and grading, were not systematically collected and thus were not included in the analysis. In addition intrinsic subtypes as defined by the PAM50 test can also predict response to anti-HER2-treatment [16], but were not available at the time point the trial was designed and conducted. This was an exploratory retrospective investigation of a prospective randomized trial and the results can be considered hypothesis generating for further de-escalating research strategies. The observations, including low HER2- copy as a negative predictive marker, arguing for antibody therapy without chemotherapy in selected patients, deserves further investigation in an independent data set. Our preliminary findings nevertheless reinforce the previously reported information [5] that de-escalation strategies may be discussed in individual patients with HER2-positive ABC.

## Conclusion

Durable clinical benefit can be achieved with trastuzumab monotherapy in a subgroup of HER2-positive patients with advanced disease and it is predictive for longer OS.

Further investigation of predictive biomarkers is needed to better characterize this subgroup of patients and inform further de-escalating strategies.

## Additional file

Additional file 1: **Table S1.** List of ethics committees (DOCX 102 kb)

## Data Availability

The data that support the findings of this study are available from the sponsor Swiss Group of Clinical Cancer Research (SAKK) but restrictions apply to the availability of these data, which were used under license for the current study, and so are not publicly available. Data are however available from the authors upon reasonable request and with permission of SAKK.

## Abbreviations

ABC: Advanced breast cancer
ALP: Alkaline phosphatase
CR: Complete response
ER: Estrogen receptor
Hb: Haemoglobin
NPF: Negative predictive factors
OS: Overall survival
PD: Progressive disease
PgR: Progesteron receptor
PR: Partial response
SAKK: Swiss Group for Clinical Cancer Research
SD: Stable disease
TTP: Time to progression
WBC: White blood cell count
