# Supplementary material for: Long-term responders to trastuzumab monotherapy in first-line HER-2+ advanced breast cancer: characteristics and survival data
Source: BMC Cancer. 2019 Sep 10;19:902. doi: 10.1186/s12885-019-6105-3 (PMC6734335; doi:10.1186/s12885-019-6105-3)
Supplement: Supplementary file 1 — Table S1. List of ethics committees (DOCX 102 kb) [file 12885_2019_6105_MOESM1_ESM.docx]

| **Ethics committees** | **Institute** | **Reference number** |
| --- | --- | --- |
| Ethikkommission Kantonsspital Aarau | Kantonsspital Aarau | None |
| Kantonale Ethikkommission Aargau | Kantonsspital Baden and private practice Dr. med. Beretta | None |
| Ethikkommission regionales Departement innere Medizin beider Basel | Universitätsspital Basel | M158/99 |
| Kantonale Ethikkommission Bern | Inselspital Bern | 111/99 |
| Kantonale Ethikkommission Graubünden | Kantonsspital Graubünden | None |
| Commission d’éthique du Département de Gynécologie et d’Obstétrique des Hôpitaux Universitaires de Genève | Hôpitaux Universitaires de Genève | None |
| Commission d’éthique de la recherche clinique de la faculté de médecine | CHUV - Centre hospitalier universitaire vaudois, Lausanne | F-92/99 |
| Kantonsspital St. Gallen Ethikkommission | Kantonsspital St. Gallen | None |
| Comitato etico cantonale Ticino | IOSI | None |
| Kantonale Ethikkommission Bern | Spital STS AG Thun | None |
| Ethik-Kommission der beiden Stadtspitäler Triemli und Waid Zürich | Stadtspital Triemli, Zürich | None |
| Ethisches Komitee Dept. Innere Medizin der Universitätsspital Zürich | Universitätsspital Zürich | EK-599 |
| Comitato Etico Ospedale di Circolo e Fondazione Macchi | Ospedale di Circolo e Fondazione Macchi, Varese, Italy | 1519 |
| Comitato Etico Istituto Europeo di Oncologia | Istituto Europeo di Oncologia, Milan, Italy | R40-IEO S40/200 |

Additional file 1
